# Supplementary material for: Identification of Deoxynivalenol and Degradation Products during Maize Germ Oil Refining Process
Source: Foods. 2022 Jun 13;11(12):1720. doi: 10.3390/foods11121720 (PMC9223215; doi:10.3390/foods11121720)
Supplement: Supplementary file 1 [file foods-11-01720-s001.zip › foods-1699413-supplementary.pdf]

Identification Deoxynivalenol and Degradation Products during Maize Germ Oil Refining Process

Yuqian Guo [1], Tianying Lu [1], Jiacheng Shi [2], Xiaoyang Li [3,*], Kesheng Wu [4] and Yonghua Xiong [1,3,*]

1 State Key Laboratory of Food Science and Technology, Nanchang University, Nanchang 330047, China;

2 Beijing Center for Disease Prevention and Control, Beijing 100013, China;

3 School of Food Science and Technology, Nanchang University, Nanchang 330047, China;

4 Jiangxi Institute of Veterinary Drug and Feedstuff Control, Nanchang 330096, China;

* Correspondence to:

Dr. Xiaoyang Li

School of Food Science and Technology, Nanchang University,

Nanchang 330047, China

E-mail: xyli@ncu.edu.cn;

Dr. Yonghua Xiong

State Key Laboratory of Food Science and Technology, Nanchang University,

Nanchang 330047, P.R. China

E-mail: yhxiongchen@163.com;

Table S1: The MS/MS data of DON and ^{13}C-DON.

Compound name	Precursor ions (m/z)	Qualitative ion (m/z)	Quantitative ion (m/z)
DON	295	138	265
^{13}C-DON	310	145	279

Table S2: The transfer rules of DON during the refining process. The conservation of matter is calculated according to each component produced in the corn germ oil processing shown in Figure 2.

Sample	Name	Concentration of DON (ng/g)	Weight (g)	Total quality of DON (ng)	Percentage (%)
S1	Crude oil	998.3	50.0	49918.7	100.00
S5	Colloidal impurities	1562.5	16.0	24999.3	50.1
-	Total difference	/	/	7617.3	15.2
S2	Degummed oil	346.0	50.0	17302.1	34.7
S6	Saponin	627.6	8.0	5020.7	10.1
S7	Wash Water	61.0	8.0	488.0	1.0
-	Total difference	/	/	10877.6	21.8
S3	Deacidified oil	18.3	50.0	916.0	1.8
S8	Clay	86.0	8.0	688.0	1.4
-	Total difference	/	/	288.0	0.4
S4	Decoloration oil	0.0	50.0	0.0	0.0

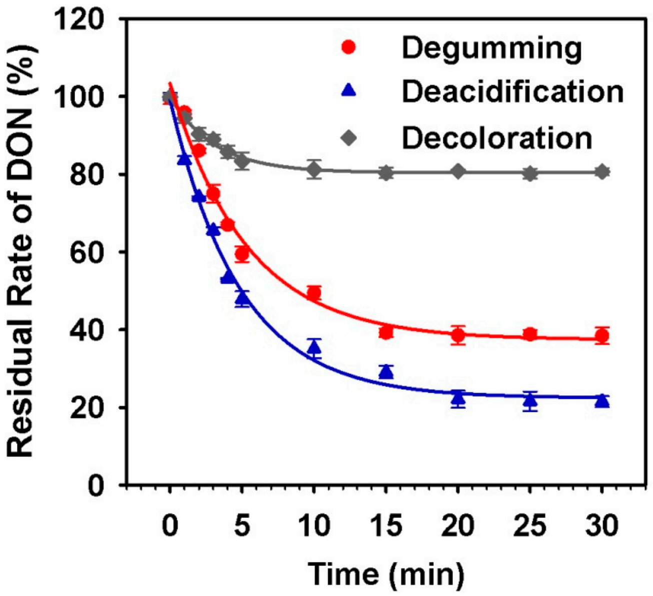

Figure S1. The variety of residual rate of DON in different refining processing from 0 to 30 minute.

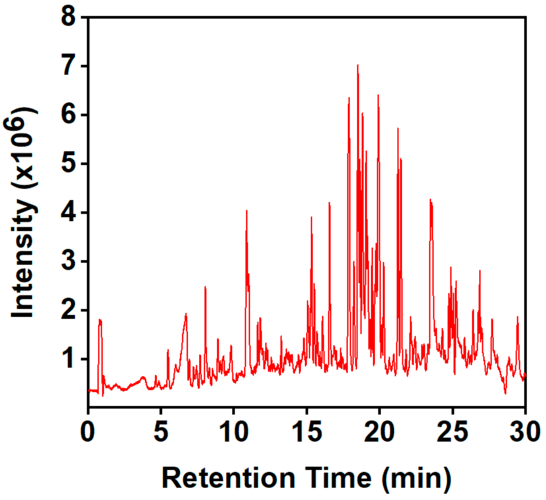

Figure S2. Total-ion chromatogram of germ oil extraction.

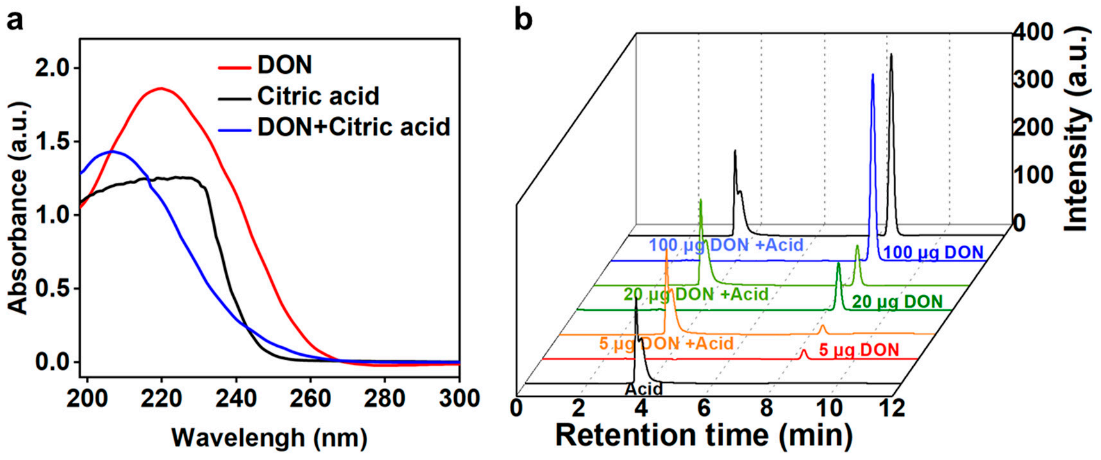

Figure S3 Characterizations of the DON variations in simulated degumming process in aqueous solution. (a) UV-vis absorption spectra of the DON or the solution of acid treatment. (b) HPLC chromatogram spectra of the different amounts DON and the solutions after acid treatment.

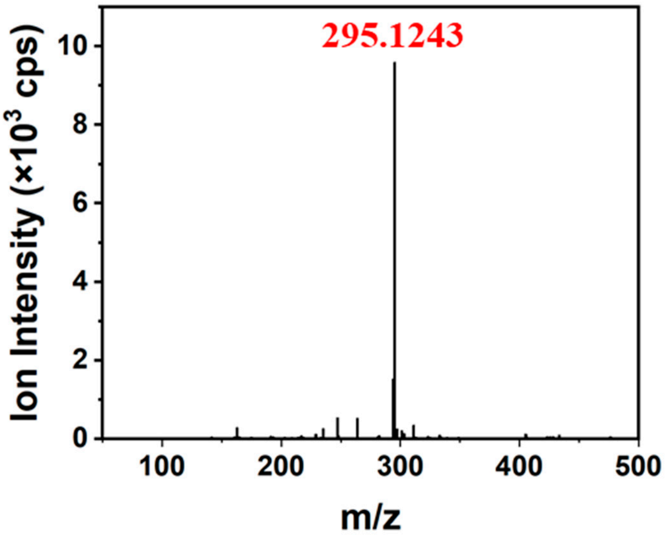

Figure S4 TOF-MS spectra of DON.

Table S3: Assignments of signals for ^{1}H NMR, ^{13}C NMR, ^{1}H–^{13}C HSQC and ^{1}H–^{1}H COSY of norDON B.

Carbon atom	δ_C	δ_H	^{1}H–^{1}H COSY	^{1}H–^{13}C HSQC
1	35.00	*ND*	*ND*	*ND*
2	66.92	3.80	H-3	+
3	72.45	4.55	H-2, H-4	+
4	14.84	2.13	H-3	+
5	55.33	*ND*	*ND*	*ND*
6	121.15	*ND*	*ND*	*ND*
7	123.04	6.79	H-8	+
8	114.33	6.67	H-7	+
9	118.09	*ND*	*ND*	*ND*
10	122.51	4.70 (10-OH)	*ND*	*ND*
11	114.33	*ND*	*ND*	*ND*
12	45.49	2.00	*ND*	+
13	21.58	1.39	*ND*	+
14	12.85	1.37	*ND*	+
2-OH	*ND*	2.71	H-2	*ND*
3-OH	*ND*	2.67	H-3	*ND*
12-OH	*ND*	2.55	*ND*	*ND*
Chemical structure of norDON B	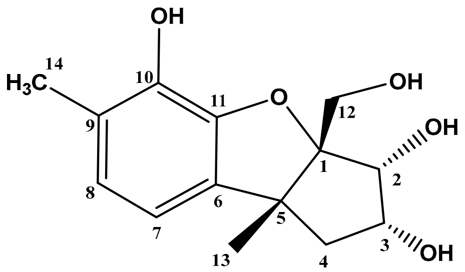			

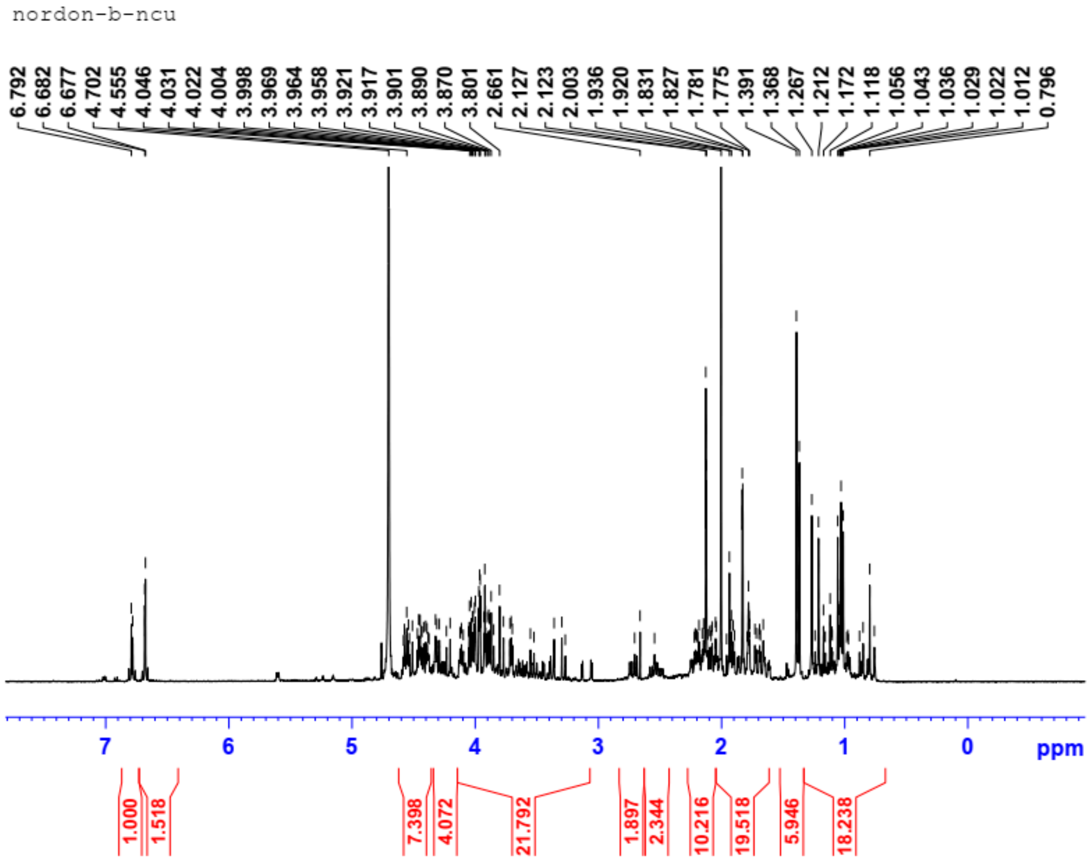

Figure S5. ^{1}H NMR spectrum of norDON B.

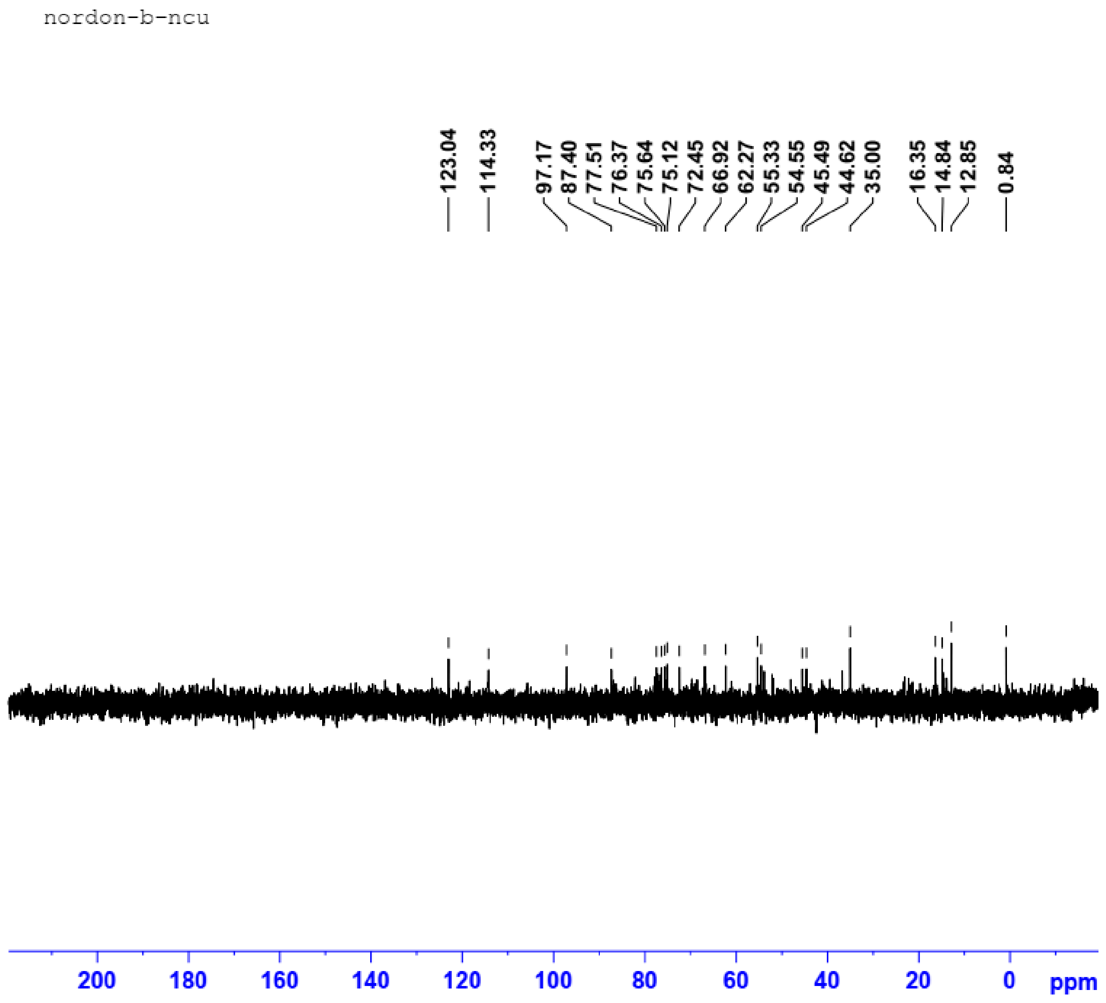

Figure S6. ^{13}C NMR spectrum of norDON B.

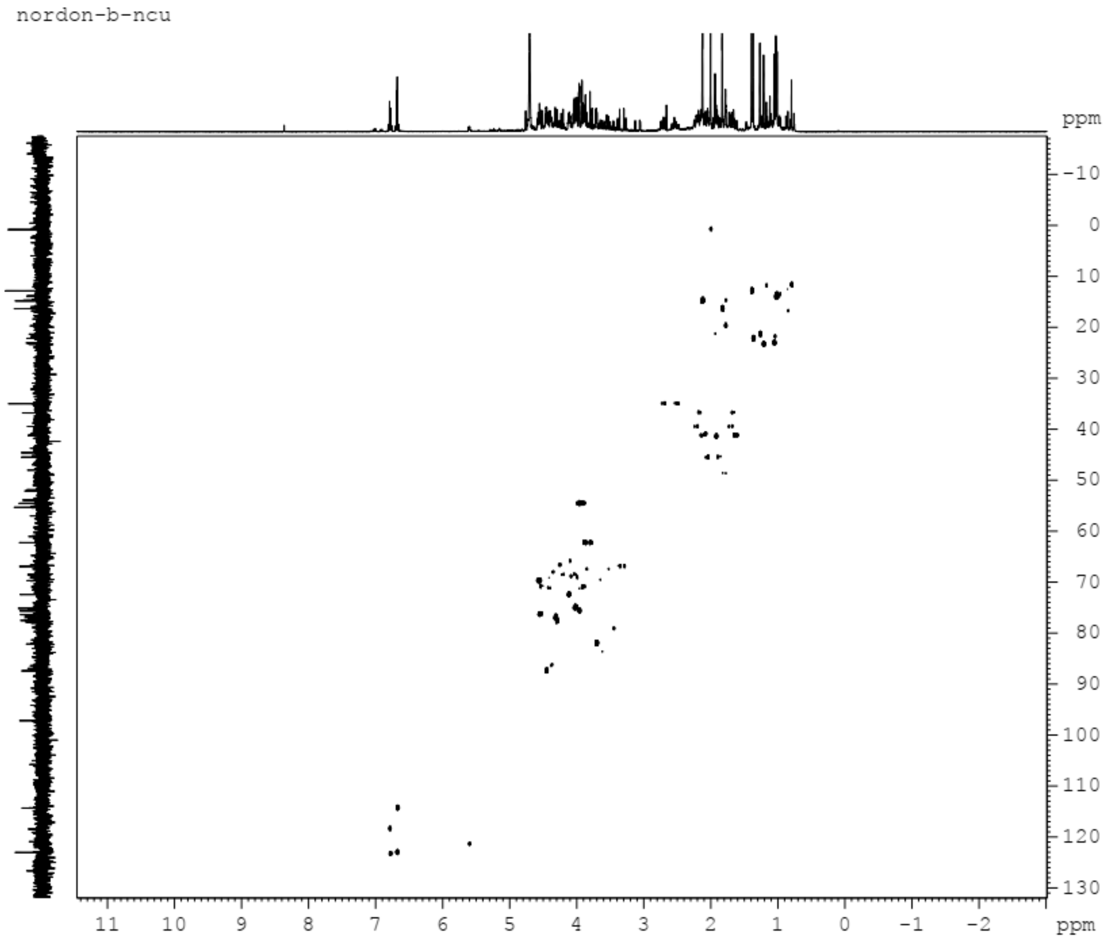

Figure S7. ^{1}H–^{13}C HSQC spectrum of norDON B.

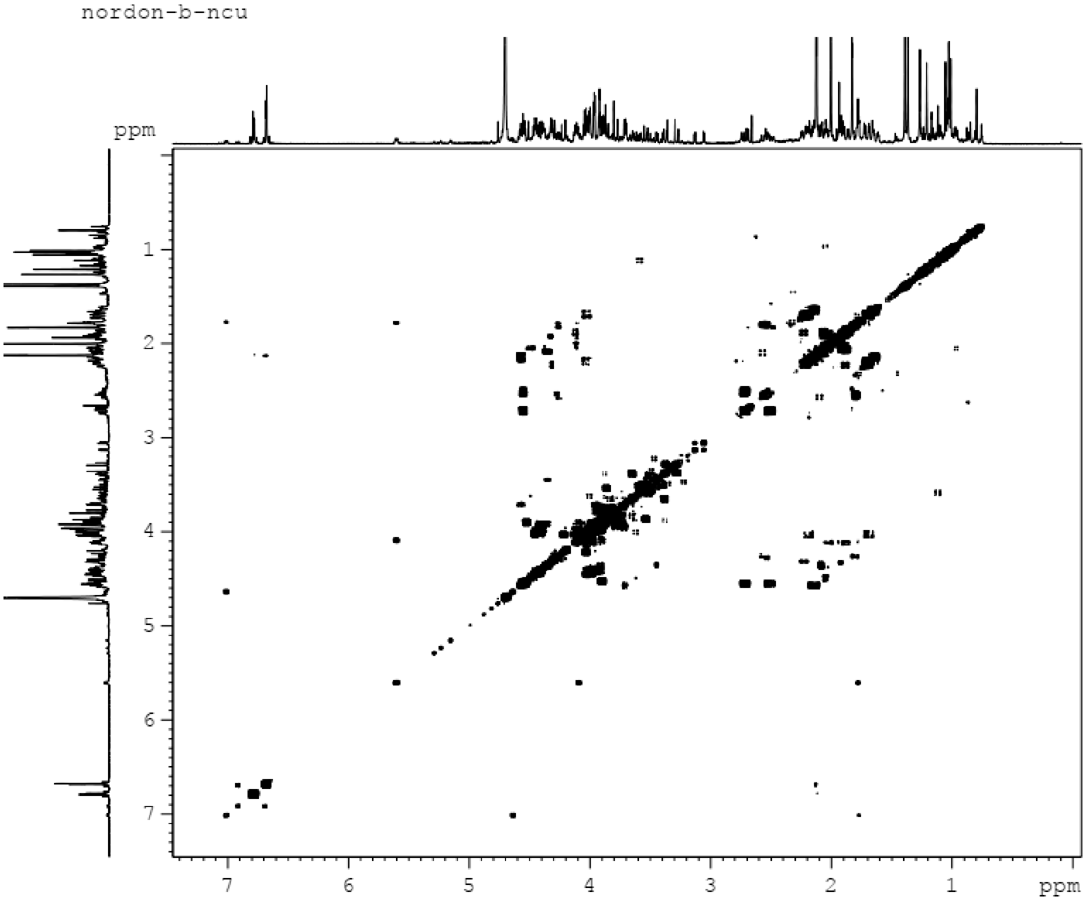

Figure S8. ^1H–^1H COSY spectrum of norDON B.

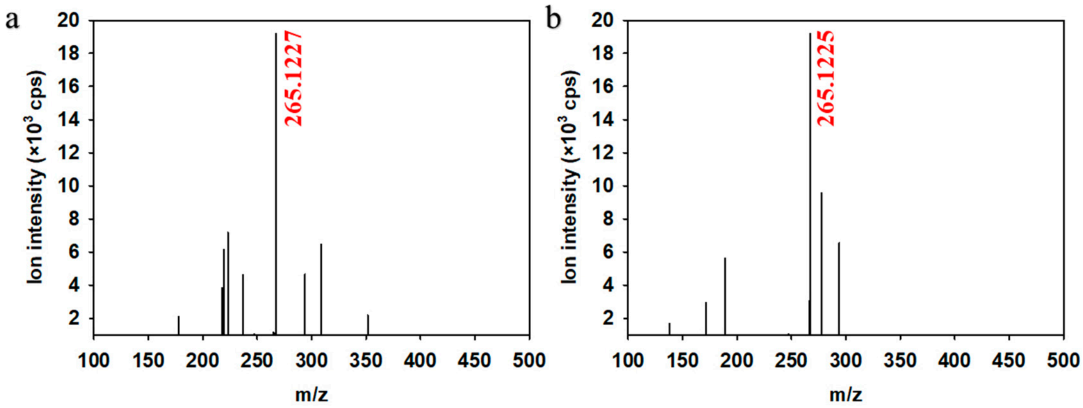

Figure S9. TOF-MS spectra of norDON B detected in saponin (a) and washing water (b).

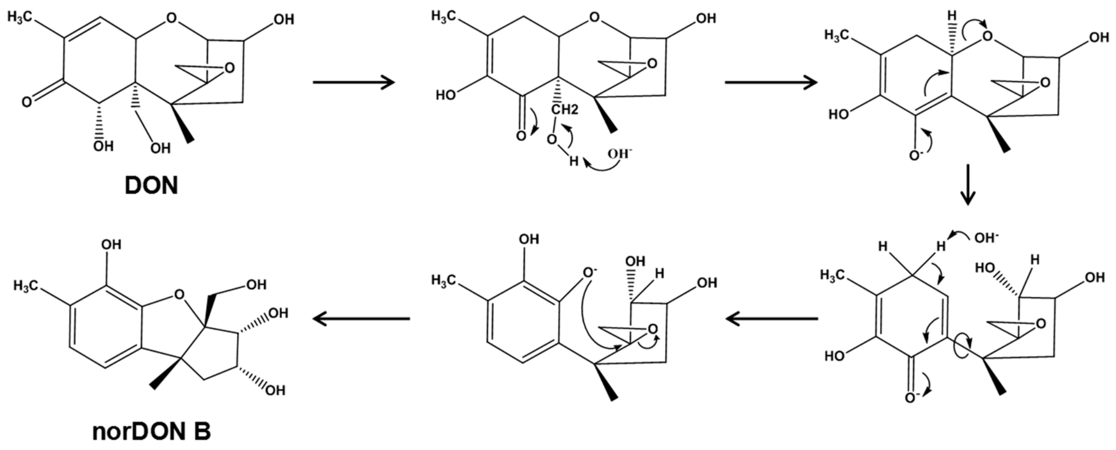

Figure S10. Potential conversion mechanism of DON during alkali neutralization refining process.
